# How to explain the beneficial effects of platelet‐rich plasma

**DOI:** 10.1111/prd.12565

**Published:** 2024-04-10

**Authors:** Reinhard Gruber

**Affiliations:** ^1^ Department of Oral Biology, University Clinic of Dentistry Medical University of Vienna Vienna Austria

**Keywords:** bone regeneration, dentistry, dermatology, fibrin, growth factors, leukocytes, orthopedics, platelet‐rich plasma, platelets, PRF, PRP, sports medicine, wound healing

## Abstract

Platelet‐rich plasma (PRP) is the platelet and leukocyte‐containing plasmatic fraction of anticoagulated autologous blood. While evidence supporting the clinical use of PRP in dentistry is low, PRP is widely used in sports medicine, orthopedics, and dermatology. Its beneficial activity is commonly attributed to the growth factors released from platelets accumulating in PRP; however, evidence is indirect and not comprehensive. There is thus a demand to revisit PRP with respect to basic and translational science. This review is to (i) recapitulate protocols and tools to prepare PRP; (ii) to discuss the cellular and molecular composition of PRP with a focus on platelets, leukocytes, and the fibrin‐rich extracellular matrix of coagulated plasma; and finally (iii) to discuss potential beneficial effects of PRP on a cellular and molecular level with an outlook on its current use in dentistry and other medical fields.

## 1 PROTOCOLS AND TOOLS TO PREPARE PRP

In the early 80s, Helene Matras, an Austrian female maxillofacial surgeon, introduced the concept of a fibrin‐rich matrix to the dental community,^1^, ^2^ followed by the work of Knighton et al. and Whitman et al., who brought the platelets into play.^3^, ^4^ It was, however, not until Marx et al. in 1998 introduced “platelet‐rich plasma” (PRP) as an autologous blood product, apparently supporting graft consolidation.^5^ The clinical success of this concept was based on two surrogate parameters, that is, bone density in the panoramic X‐rays and from morphometry of bone biopsies.^5^ Less known is how the PRP was produced. In total, 400–450 mL blood, anticoagulated with citrate phosphate dextrose, was subjected to two sequential centrifugations, coming up with three fractions, that is, namely, 70 mL PRP, 200 mL platelet‐poor plasma (PPP), and 180 mL erythrocyte fraction. PRP showed a threefold enrichment of platelets compared to whole blood. While PPP and erythrocytes were returned to the patients, PRP was available for the treatment, initially to prepare a PRP gel falling under the category of solid PRP. Aliquots of PRP were mixed with 1% CaCl_2_ and 100 U bovine thrombin/mL until a PRP gel was formed. The authors stated that “Once the PRP is added to the graft, the fibrin formation binds the otherwise loose cancellous cellular marrow together to assist the surgeon in sculpting the graft.”^5^ They were already proposing the concept of the sticky bone. Thus, it was an automated system established in transfusion medicine and blood banks that should become the basis of future PRP protocols.

The double‐spin or two‐step centrifugation protocol was proposed as a standard PRP preparation method. Anticoagulated blood's first “soft” spin separates the yellow plasmatic fraction containing the platelets and leukocytes from the heavier red erythrocyte fraction. This yellow plasmatic portion, containing the leukocyte‐rich buffy coat layer, is then transferred to a new tube or chamber. The second “hard” spin allows platelets and leukocytes to form a pellet at the bottom of the tube or chamber. Next, at least half of the cell‐free PPP is discarded, and the pellet is resuspended in the remaining plasma. This method results in an approximately fivefold enrichment of platelets, equivalent to 1 million platelets/μL. Depending on whether or not the buffy coat layer is included in the second “hard” spin, leukocytes are enriched in PRP or not. The PRP can now be applied as liquid PRP or by adding CaCl_2_ and, if required, thrombin to push the coagulation as solid PRP, which is a PRP gel.

Two‐step centrifugation protocols were developed based on this concept. Disposable two‐chamber systems, for instance, the PCCS method (PCCS Kit, 3i Implant Innovations, Palm Beach Gardens, FL, USA) and the Smart PReP system (Harvest Technologies Corporation, Munich, Germany) were introduced. Both systems require 60 mL of blood to generate around 7 mL of liquid PRP. Alternatively, various “open” tube‐based systems have been presented that require uncapping off the tubes and transferring fractions by pipetting. To overcome this limitation, “closed” two‐chamber systems, designed to be used in a regular centrifuge with swing‐out rotors, have been introduced. Here, 50 mL of anticoagulated blood is separated by a first “soft spin” into the erythrocyte fraction, the buffy coat, and PPP. The innovation here is that the erythrocyte fraction, with or without the buffy coat, can be separated from the plasmatic part by an adjustable knob screw mechanism. Using the same tube, a second “hard spin” generates the pellet accumulating platelets and leukocytes at the surface of the knob. Following the principles of cell enrichment, PPP is partially withdrawn, and the pellet is resuspended in the remaining volume. If required, coagulation is initiated. Trademarks of these devices are, among others, Dr. PRP® and TubexPlus®.

One‐step centrifugation protocols became available, including variations of the classical tubes containing a separation gel and anticoagulants. The gel migrates, forming a rigid barrier between the plasma containing platelets and binding leukocytes and erythrocytes. The one‐step liquid PRP may be aspirated directly from the tube or further concentrated to increase the platelets in a second hard spin. These protocols are proposed by various manufacturers, including Tropocells®, claiming enrichment of platelets and monocytes, while neutrophils and erythrocytes are sequestered within and beneath the gel barrier. Also, Hanbaihan Medical Devices Co., Ltd. offers a series of PRP tubes with separation gel with and without anticoagulants. The composition of the separation gels is of research interest, aiming to increase the recovery rate of platelets.

The Endoret System (BTI Biotechnology Institute, S.L., Spain) also exemplifies the one‐step protocol. Anticoagulated blood is separated at 580 g for 8 min in a glass tube.^6^ The PRP fraction is collected by avoiding the buffy coat layer, thus preparing a leukocyte‐poor PRP. With an approximate twofold platelet enrichment, the PRP fraction can be used as liquid PRP, termed “Plasma Rich Growth Factor or PRGF®,” or coagulated by adding CaCl_2_. The PRP fraction can be separated into the retracted fibrin‐rich gel and the respective serum fraction.^7^ The serum fraction used for various clinical applications, for instance, in ophthalmology,^8^ consists of molecules released from the twofold enriched activated platelets and other serum components. Endoret System also introduced a product where CaCl_2_ can be omitted.

In summary, when preparing liquid PRP, we can differentiate between the two‐step and one‐step centrifugation protocols of anticoagulated blood. Another variation in preparing PRP is whether or not the buffy coat possibly “contaminated” with some erythrocytes is included in the preparation—if yes, this is a “leukocyte‐rich PRP.” Alternatively, if only the upper layer immediately adjacent to the buffy coat is harvested, this is a “leukocyte‐poor PRP,” where platelet yield is also reduced. Independent of the protocol, if coagulation is required, CaCl_2,_ or a combination with thrombin, is added. The solid PRP or PRP gel consists of the retracted fibrin‐rich matrix where platelets and leukocytes are entrapped, and the respective serum fraction. Thus, the basic concept is the centrifugation of anticoagulated blood and, later, the injection of the liquid PRP to the defect site. However, this concept has not been proven to be superior to the local injection of autologous whole blood, for instance, to treat elbow or Achilles tendinopathy,^9^, ^10^ lateral epicondylitis,^11^, ^12^ or plantar fasciitis^13^ with even historical attempts in the treatment of subconjunctival autologous blood injections.^14^ The clinical success of autologous whole‐blood injections has to be considered in the attempt to explain the beneficial effects of PRP injections.

## 2 CELLULAR AND MOLECULAR COMPOSITION OF PRP

The cellular compositions of PRP depend on two variables: (i) whether a one‐step or two‐step centrifugation protocol has been chosen and (ii) whether the leukocyte‐rich buffy coat is part of the preparation. Other variables include the “relative centrifugation force (RCF),” centrifugation time, and selection of the rotor that needs to be optimized depending on the tubes or chamber system. The overall aim is to maximize the platelet recovery rate, also considering the presence of leukocytes. For instance, the PCCS method generats PRP with a median of about 2 200 000 platelets/μL,^15^ and the Smart PRePTM system 1 200 000 platelets/μL PRP.^16^ The average number was 280–290 000 platelets/μL blood.^15^, ^16^ As a rule of thumb, the two‐step protocols should reach an approximately fivefold enrichment of platelets. One‐step protocols will reach an approximately twofold enrichment of platelets.

It is important to note that, for instance, PCCS and Smart PRePTM accumulate about 15–20 000 leukocytes/μL PRP—which is roughly twofold enriched in leukocytes considering 6–7000 leukocytes/μL blood.^15^, ^16^ Within the PRP‐leukocyte fraction, a distribution of approximately 70% lymphocytes, 20% monocytes, and 10% neutrophils was reported.^17^ Others found 23% neutrophils and reported around 12 000 lymphocytes/μL PRP and 4000 neutrophils/μL PRP.^18^ The normal range is between 3000 and 9500 lymphocytes/μL and between 2500 and 7000 neutrophils/μL blood. Thus, lymphocytes, but not necessarily the heavy neutrophils, are enriched in PRP when compared to blood.^19^ In transfusion medicine, leukocyte‐depleted platelet concentrates are prepared using the apheresis technique and from the buffy coat. The leukocytes are trapped in a filter.^20^ Erythrocytes are separated, and platelets and leukocytes are enriched in the buffy coat. Essentially, this PRP is a platelet and leukocyte‐rich plasma.

The molecular composition of the plasma fraction of PRP is relatively similar to blood. Among the significant dissolved proteins are serum albumins, globulins, fibrinogen, and—essential for coagulation—a series of clotting factors. Thus, PRP is a suspension of enriched platelets and leukocytes in anticoagulated plasma. Theoretically, centrifugation of anticoagulated blood does not activate platelets and leukocytes, which is wrong as platelets are stress‐sensitive.^21^ Nevertheless, when liquid PRP is injected into a defect site, we assume that under these conditions, citrate is diluted and the natural process of coagulation is initiated, including platelet activation and thus the release of growth factors and other signaling molecules, as described later, presumably initiate or at least support local healing events. However, we should not rule out that when PRP is injected into a low‐blood flow area such as knees, the dispersion of citrate may not be as effective, and platelet activation is delayed.

In summary, the significant difference between the regular blood clot and PRP is the presence or absence of the erythrocytes, while the other cell types—platelets and leukocytes—are enriched. Another significant difference is that while regular blood clots undergo spontaneous coagulation, liquid PRP needs to be activated either ex vivo by CaCl_2_ or in vivo upon injection at the defect site. Growth factors and other signaling molecules are higher in PRP than in the regular blood clot; however, explaining PRP effects simply by the enriched platelets is presumably an oversimplification of the therapeutic concept.

Surprisingly, the role of erythrocytes in the course of wound healing remains unclear. Only indirect evidence suggests that erythrocytes lower the biomechanical properties of the blood clot^22^ and modulate fibroblast proliferation, collagen production, and scaffold contraction in vitro.^23^ Moreover, erythrocytes have large amounts of catalase, a pivotal enzyme that defends against oxidative stress,^24^ more than activated platelets.^25^, ^26^ Finally, hemorrhagic erythrocytes are likely removed by macrophages^27^ when granulation tissue begins to invade the wound space approximately 4 days after injury.^28^ Erythrophagocytes are essential to prevent the extracellular release of toxic hemoglobin and heme^29^; the macrophages become pro‐resolving and support wound healing.^29^, ^30^ Therefore, hematoma resolution is an integral early step in wound healing. If erythrophagocytosis positively impacts the early healing event, a certain proportion of erythrocytes in PRP might be beneficial when preparing PRP.

## 3 ANTICOAGULANTS AND THE EFFECTS OF THROMBIN AND CaCl_2_

Clinically, thinking about thromboembolic disease, anticoagulation is the prevention or reduction of the progression of myocardial infarction,^31^ ischemic stroke,^32^ hypertension,^33^ or pulmonary embolism,^34^ to name a few. Drugs inhibit platelet aggregation or specific pathways of the coagulation cascade, blocking the formation of fibrin and stable platelet aggregates. Warfarin and dicoumarol block the recycling of vitamin K, which would be required for the carboxylation and, thus, activation of coagulation factors, including prothrombin.^35^ Heparins work mainly by activating antithrombin III, which in turn inactivates coagulation factors, including thrombin and factor Xa.^36^ Today, we have directly acting oral anticoagulants (DOACs), including a thrombin inhibitor (dabigatran) and factor Xa inhibitor (rivaroxaban, apixaban, betrixaban, and edoxaban).^37^ These strategies are critical for thromboembolic disease but not for preparing PRP.

In transfusion medicine, citrate prevents blood from coagulation, based on the fundamental discoveries of Olof Hammarsten dating back to 1875.^38^ Citrate binds to free calcium and prevents it from interacting with the coagulation system.^38^ A ratio of three citrate molecules to one of calcium is necessary to prevent clotting. Usually, 1 mL of 3.8% sodium citrate is mixed with 9 mL of blood to prevent its coagulation. Recalcification and, thus, coagulation of PRP can be achieved by reaching a final concentration of 0.5% CaCl_2_, ideally in rough surface glass tubes that support coagulation. Apart from its anticoagulation properties, citrate in PRP may have a positive impact on bone regeneration, at least when interpreting the findings observed with citrate‐based polymer/hydroxyapatite^39^ or calcium citrate alone,^40^ and the potential of citrate to inhibit bone‐resorbing osteoclasts.^41^ Even in wound healing formulas, panthenol citrate or sildenafil citrate is used.^42^, ^43^ In volunteer apheresis blood donation procedures, however, repeated citrate exposure is supposed to affect calcium homeostasis, accumulating in overall bone loss.^44^ However, well‐designed studies on male volunteer blood donors do not support this hypothesis.^45^

Thrombin speeds up spontaneous coagulation. While it was initially purified, natural bovine thrombin possibly caused adverse reactions.^46^ Thrombin from bovine resources is critical^47^ because it can develop antibodies, usually against factor V/Va, which may lead to hemostatic abnormalities.^48^ Antibodies may also arise from contact with antigenic sources other than bovine‐derived thrombin.^49^ Alternatively, autologous serum or fractions are mixed back into the citrated PRP to support the coagulation cascade.^50^ Otherwise, recombinant thrombin or protease‐activated receptor agonists are used.^51^ Thrombin then converts soluble fibrinogen of the plasma into insoluble strands of fibrin^52^ and catalyzes other coagulation factors, including the XIII, to stabilize the fibrin clot.^53^ Thrombin further promotes platelet activation and aggregation via the cleavage of protease‐activated receptors on the cell membrane of the platelet,^53^ as described later in this review.

Taken together, even though citrate cannot prevent platelets and leukocytes from being at least moderately activated during blood centrifugation,^21^ PRP remains liquid and ready to be injected into a defect site. There, the citrate is diluted, and coagulation of the plasmatic components is initiated. Platelets and leukocytes, together with the fibrin‐rich matrix, are further activated, forming a kind of internal blood clot, similar to hemorrhage, where blood leaks from damaged blood vessels. In the case of PRP, however, this localized and therapeutic “bleeding” does not turn red because of the lack of erythrocytes. Anticoagulants simplify the handling of blood and reduce unwanted platelet activation while preparing PRP. Hence, liquid PRP holds the plasma components and platelets ready to release their “cargo” into the required side.

## 4 BIOACTIVE MOLECULES RELEASED FROM ACTIVATED PLATELETS AND OTHER CELLS

Once activated, platelets release the granules' content by exocytosis.^54^ Platelets contain at least three major types of granules—α‐granules, dense granules, and lysosomes. Platelets carry 50–80 α‐granules.^55^ They contain mainly proteins, both membrane‐associated receptors (e.g., αIIbβ3 and P‐selectin) and soluble proteins involved in hemostasis (e.g., von Willebrand factor and factor V), inflammation (e.g., chemokines such as CXCL1, CXCL4, CXCL5, CXCL7, CXCL8, CXCL12, CCL2, CCL3, and CCL5),^56^ and wound healing (e.g., platelet‐derived growth factor, vascular endothelial growth factor, and fibroblast growth factor), and antimicrobial responses^57^—all together showing up in platelet releasate proteomics.^58^ Platelets also carry 3–8 dense granules containing bioactive amines (e.g., serotonin and histamine), adenine nucleotides, polyphosphates, and pyrophosphates, as well as high calcium concentrations.^57^ Lysosomes contain acid hydrolases and proteases. Furthermore, T‐granules have been identified as carrying, for instance, TLR9.^59^ Activated platelets also release exosomes and microparticles.^57^ Therefore, liquid PRP is a rich source of platelets that are ready to release the content of the granules, ideally upon natural activation at the injection site.

When preparing PRP, the buffy coat is the fraction of uncoagulated blood that contains most of the white blood cells. However, blood banks also use the buffy coat to prepare platelet concentrates.^60^, ^61^ Leukocytes or white blood cells that help the body fight off infection are 10‐20x more concentrated in the buffy coat than in the whole blood.^62^ Apart from platelets, the buffy coat consists of lymphocytes, T cells, B cells, and natural killer cells that play a vital role in the immune system. Monocytes help in the immune response by isolating and digesting harmful microorganisms. Monocytes can differentiate into macrophages, dendritic cells, or even osteoclasts. Granulocytes can be divided into basophils, eosinophils, and neutrophils, which secrete chemicals, attack parasites, and kill bacteria/fungi. It cannot be easily summarized what exactly every cell type contributes to regeneration, but overall, all cell types are supposed to add to inflammation and its resolution, as well as wound healing and bone regeneration.

It is at the level of speculation whether or not a one‐shot of slightly supraphysiological number of leukocytes supports or even impairs wound healing, considering that particularly neutrophils and later on monocytes infiltrate a wound healing site anyhow; however, when PRP is injected, for instance, to treat tendon, muscle, and ligament injuries, PRP is not the better equivalent of a blood clot, it is the stand‐alone therapy. The extra platelets and leukocytes may support cellular events that help wound healing and bone regeneration. However, in chronic wounds, excessive neutrophil activity can increase the amount of damage and prolonged inflammation and ultimately hinder proper healing.^63^, ^64^ Hence, it is impossible to give a simple answer that considers the complex pathophysiology of the various defect sites and types of injuries to potentially benefit from PRP injections. Mouse models supporting the critical role of monocytes, for instance, for wound healing^65^ and bone regeneration,^66^ are only part of this mosaic of information we need to get closer to a personalized medicine approach for PRP therapy.

## 5 WHAT IS THE ROLE OF THE FIBRIN‐RICH ECM DURING HEALING AND REGENERATION?

The role of fibrinogen and fibrin in hemostasis and thrombosis,^67^ as well as their formation, structure, and properties, is an area of intensive research, as illustrated recently.^68^ In brief, fibrinogen (factor I) is produced in the liver and is a main component of the plasma after the initiation of the coagulation cascade; it is thrombin, which cleaves the N‐terminus of the Aα and Bβ chains in fibrinogen. The fibrinopeptides released are active; for instance, fibrinopeptide A induces the expressions of inflammatory cytokines in vascular smooth muscle cells.^69^ The nascent fibrin strands polymerize and are later crosslinked by XIIIa, resulting in a fibrin meshwork.

The fibrin meshwork is further reinforced by the activated and aggregating platelets being sticky through GpIIb/IIIa.^68^ Thus, platelets support the consolidation of the fibrin‐rich matrix; apart from serving as a source of growth factors and other signaling molecules, platelets stabilize the fibrin matrix.^68^ This matrix helps hemostasis and provides a scaffold allowing neutrophils, monocytes, fibroblasts, and blood capillaries to repopulate the defect site. The binding and migration of cells into a fibrin matrix require fibronectin and vitronectin, both of which are cell‐adhesive glycoproteins. For instance, fibronectin provides a conduit for fibroblast transmigration from the collagenous stroma into the fibrin matrix.^70^ It is thus not only fibrin but also other plasma proteins that support cells immigrating the fibrin‐rich matrix originating from blood and PRP.

Fibrinolysis, as illustrated recently,^71^ allows cells to repopulate the fibrin‐rich matrix, a process strictly dependent on activating the plasminogen, a liver‐born protease intrinsic to blood. For instance, bone regeneration depends on plasminogen and fibrinolysis, not necessarily on the presence of fibrinogen, at least in mouse models.^72^, ^73^ Plasminogen is activated by tissue plasminogen activator and urokinase plasminogen activator being expressed by immigrating cells. The local plasmin‐based fibrinolysis allows the immigration of cells into the fibrin‐rich matrix. The platelet secretome, for instance, can activate the fibrinolytic activity of mesenchymal cells.^74^ Plasmin is a pleiotropic protease linked to activating collagenase and the complement system, but here, the fibrinolytic activity is the focus.

In this context, it is interesting to speculate about the role of fibrinogen, fibronectin, and the other plasma components upon injecting liquid PRP into a defect site. Under the premises that platelets are activated by the tissue factor (factor III, or CD142), a cell surface glycoprotein that makes a complex with VIIa, or the VIIIa:IXa complex of the intrinsic pathway, all routes to generate Xa and thrombin, suggesting that liquid PRP undergoes coagulation “in‐tissue” and the resulting fibrin‐rich matrix has a retard function for platelet‐ and leukocyte‐derived signaling molecules. Concerning the topical application of PRP gels, the concept of the “healing blastema” can be extended toward serving as a provisional fibrin‐rich matrix to protect the wound toward a septic environment while allowing the natural healing process to take place—recapitulating the conserved sequence of wound healing^28^ and bone regeneration.^75^

## 6 POTENTIAL BENEFICIAL EFFECTS ON A CELLULAR AND MOLECULAR LEVEL

When using PRP, we assume that the enrichment of platelets and their release of growth factors and other bioactive molecules can positively influence wound healing and bone regeneration. According to the motto, the more platelets the better the healing; studies on the tenant gene effects of platelets and the growth factor are interpreted positively in vitro. Also, the enrichment of the white blood cells is positively represented in that the physiological meaning of innate immunity is emphasized. Convincing preclinical experiments showing a relationship between the number of platelets and leukocytes in the plasma with wound healing and bone regeneration parameters still need to be conducted. It is wise to go one step back and ask, “What is the role of the hematoma in bone regeneration?”^76^ The hematoma is essential because its removal during surgery impairs fracture healing.^76^ This early fracture hematoma is rich in granulocytes, monocytes/macrophages, and lymphocytes—and the cells produce an environment rich in cytokines and chemokines supporting the early immune defense.^77^ Currently, the exact role of one or another cell population of the blood clot or a PRP in the orchestrated process of early wound healing and bone regeneration is unclear, but mouse models provide some insights; for instance, that depletion of macrophages causes impaired wound healing^65^ and bone regeneration.^78^ Similar studies with depletion of platelets have not been performed so far—but in vitro studies suggest an activity of platelets to support cellular healing events.

In vitro research provides the bioassay reflecting what is known from the platelet releasate or secretome proteomics.^58^ For instance, we have shown that the secretome of leukocyte‐depleted and thrombin‐activated platelets is highly mitogenic and chemotactic for periosteal cells,^79^ bone‐derived cells,^80^, ^81^ and endothelial cells, also forming tiny capillaries.^82^ The platelet secretome supports osteoclastogenesis,^83^ rapidly neutralizes cytotoxic amounts of hydrogen peroxide,^25^ and increases fibrinolytic activity in mesenchymal cells.^74^ Some of these activities are classical responses to platelet‐derived growth factor isoforms, all mitogens, and chemotactic factors released by the platelets.^79^, ^80^, ^81^ Platelets also release catalase, being effective in removing hydrogen peroxide.^25^ Thus, it is not surprising that in vitro research related to fractionated blood focuses on the mitogenic and chemotactic activity of the respective preparations.^84^ However, most notably, fractionated blood can decrease osteoclastogenesis,^85^ holds anti‐inflammatory properties for macrophages^86^, ^87^, ^88^ and mesenchymal cells,^89^ and has effects that are at least partially caused by the lipid fraction^90^—similar to blood.^91^ Genomic screening essays further revealed the complexity of cell responses to fractionated blood, highlighting the role of TGF‐β signaling in fibroblastic cells^92^, ^93^, ^94^—consistent with TGF‐β confirmed by the respective proteomic analysis.^92^

It would be easy to repeat and further review other potential beneficial effects of platelets, leukocytes, and the fibrin‐rich matrix enriched with other plasma proteins, not to forget the potent biological activity of plasma lipids. However, this list of potential activities does not explain the effects of PRP or even the regular blood clot, the hematoma, on a cellular and molecular level. All the evidence we have on how PRP exerts its function is indirect and only indicative, not causal—but this biological aspect may be less important if PRP shows robust clinical success in specific applications.

## 7 CURRENT USE IN DENTISTRY AND OTHER MEDICAL FIELDS

Let us consider the reviews published in the previous year. We can see the following medical applications that include but are not limited to osteoarthritis of the knee osteoarthritis,^95^, ^96^ ankle osteoarthritis,^97^ and thumb carpometacarpal joint.^98^ PRP is also used to treat tendinopathy of the achilles,^99^, ^100^ the patella,^101^ and tennis elbow.^102^ Moreover, shoulder pathologies,^103^, ^104^ including rotator cuff disease,^105^ spinal diseases,^106^, ^107^ and surgery,^108^, ^109^ are linked to PRP treatment. Traditionally, PRP was used in chronic wound management,^110^ including diabetic foot ulcers.^111^ PRP is widely applied in skin and plastic surgery^112^ and facial rejuvenation.^113^ Treatment of alopecia became an essential application of PRP,^114^, ^115^, ^116^ as well as dermatological disorders.^117^ Also mentioned should be the application of PRP to treat urogynecology disorders,^118^, ^119^ meniscus repair,^120^ tonsillectomy,^121^ and anal fistula.^122^ PRP is mainly applied in fields outside of dentistry.

When focusing on dentistry reviews, PRP might offer some beneficial effects on clinical and radiographic outcomes for regeneration of periodontal intrabony defects.^123^ Other reviews, including ours, suggest that in oral and maxillofacial surgery, PRP is a potential strategy to improve soft‐ and hard‐tissue regeneration, with a focus on alveolar ridge preservation, management of post‐extraction complications, bone augmentation, and temporomandibular joint disorders. Seven studies showed superior outcomes when PRP was added during sinus floor elevation, and five showed no superior outcome. Three studies found a significant advantage of PRP for alveolar bone regeneration, and another three found it beneficial for soft tissue healing. Three studies reported on the beneficial effects of PRP directly during implant placement, while another study failed to find significant differences.^124^ The effect of PRP on the survival of dental implants in sinus‐augmented sites is uncertain.^125^, ^126^ However, the composition and methodology used to prepare the PRP should be a critical point when evaluating its efficacy.^124^, ^127^ Promising data further support the use of PRP in temporomandibular disorders' management effectively in reducing pain and joint sound and improving mandibular motion.^128^, ^129^ Moreover, PRP's impact on accelerating orthodontic tooth movement remains debatable, but at least three studies found a positive correlation between PRP injection and tooth movement acceleration.^130^ PRP was also used to treat oral lichen planus.^131^ There is potential for clinical use of PRP, particularly the concept of injectable PRP rather than the local use of coagulated PRP gels.

## 8 FUTURE ASPECTS AND LIMITATIONS

The leading tenor of many PRP reviews is that more controlled studies are needed. Also, the processing of PRP with the respective quality control should be standardized. These limitations are, without any debate, a weakness and a claim for future research. However, we can also discuss the limitations on a more fundamental level, more provocative asking: Do we need to fractionate anticoagulated blood anyhow to prepare an injectable autologous therapy? When we remove the erythrocytes, are we aware that they might benefit wound healing? If we consider the platelets enriched in PRP the primary source of healing activity, do we know the underlying pharmacodynamics? What is the role of the leukocytes when injected into an inflamed site, and is there a clinical impact? Questions like these could drive future research, including clinical applications.

Today's knowledge is mainly based on descriptive research; PRP is applied, and the endpoints are usually compared against “no treatment.” The explanations on the possible beneficial effects of PRP on a cellular and molecular level are typically based on findings from in vitro studies, overall supporting the fundamental knowledge that, for instance, activated platelets release a cocktail of molecules, some of which are highly mitogenic and chemotactic for fibroblasts and other cells of the mesenchymal lineage. The platelet secretome also possesses an angiogenic and anti‐inflammatory activity. Nevertheless, these in vitro findings are mainly confirmatory and are indirect evidence but no explanations for how PRP and related blood fractions exert their activity in vivo. From a clinical perspective, this lack of cellular and molecular information is acceptable as long as PRP exerts its supposed therapeutic activity, but curiosity remains.

Today's single‐cell RNAseq technology provides insights into the presumably significant changes in the transcriptome of the various cell populations at PRP injection sites, helping us understand the critical target cells and their response to PRP. This technology would also help to explain the in vivo cell response to local injection of PRP and autologous whole blood in tendinopathy,^9^, ^10^ epicondylitis,^11^, ^12^ and fasciitis.^13^ As already discussed earlier, erythrocytes might be beneficial during regeneration. Another question is whether ex vivo platelet activation is necessary for liquid PRP's clinical effect. Hypothetically, activation of platelets in liquid PRP is not mandatory as, after injection into the defective site, the protective effect of citrate falls away, and the sensitive platelets are activated locally. However, this assumption should still be confirmed experimentally. Thus, it would be interesting to revisit PRP and ask more fundamental questions rather than speculating on how many platelets are necessary to achieve the desired outcome of a particular therapeutic application.

Maybe it is an oversimplification, but therapies that work to benefit the patients, mainly when they have almost no side effects and are at low costs, are suitable—even though the underlying cellular and molecular mechanisms remain to be discovered—in a top‐down research approach. We should be excited about the research and consider today's limitations as the challenges and inspiration for future research.

## CONFLICT OF INTEREST STATEMENT

Reinhard Gruber declares no conflict of interest.

## Data Availability

Data is available on request from the authors.
